# An Evaluation of a Behaviour Assessment to Determine the Suitability of Shelter Dogs for Rehoming

**DOI:** 10.4061/2010/523781

**Published:** 2010-02-24

**Authors:** A. H. Poulsen, A. T. Lisle, C. J. C. Phillips

**Affiliations:** ^1^Centre for Animal Welfare and Ethics, School of Veterinary Sciences, The University of Queensland, Queensland 4343, Australia; ^2^School of Animal Studies, Gatton Campus, The University of Queensland, Queensland 4343, Australia; ^3^School of Land, Crop and Food Sciences, Gatton Campus, The University of Queensland, Queensland 4343, Australia

## Abstract

We evaluated a scheme for assessing shelter dog behaviour, which used 28 tests and rated responses from 0 (positive response) to 5 (fear, tonic immobility, or escape attempts). The assessment was evaluated for 236 dogs, and was repeated by a different assessor for 39 dogs approximately 80 days after rehoming to determine relevance of individual test components. A new owner survey evaluated satisfaction with the dog. A total of 130 of 236 dogs passed (score ≤ 70), 24 scored 71–80 (referred for behavioural modification), and 82 (score > 80) failed. Scores were mainly unaffected by dog type and environmental variables, but decreased if dog faeces from a previous test was present in the arena during a test. Shelter tests only correlated with repeat tests if there was no direct contact with assessors. Adopters were satisfied with their dogs, despite reporting some behaviour problems. The shelter assessment was therefore robust against most outside influences but did not predict responses to people well.

## 1. Introduction

Dog behaviour assessments in shelters are increasingly used to determine their suitability for rehoming, and help to increase the rate of successful adoptions [1, 2]. There have been few evaluations of the efficacy of the assessments, and in particular there is a need to link shelter behaviour assessment results with postadoption behaviour, as this would indicate their effectiveness in detecting dogs that are undesirable or unsafe to offer for adoption, as well as allowing refinement of the tool [1, 2]. Such examinations of the reliability of retests of dog behaviour are particularly rare [2], but a recent study found significant correlations between a test and retest 40 days later [3]. None have evaluated the repeatability of individual components of the assessment. 

When dogs are relinquished by their owners to shelters, problem behaviours such as hyperactivity, separation anxiety, and vocalisation are most often cited as the reason [1, 4, 5]. Behaviour, and in particular aggression, is also the single most common reason for dogs to be returned by new owners to shelters [6]. However, the prevalence of aggression may not be accurately reported by relinquishing owners if they are aware that aggressive dogs do not have the ability to be rehomed through many shelters [1], as well as presenting a danger to any new owner following rehoming. Within the shelters, aggression to other animals is also one of the most common behavioural problems [5]. Not all aggression is detected, for example, an American study found that 41% of dogs who passed a behavioural assessment and were subsequently adopted from an animal shelter exhibited aggression post adoption [7]. This may cause owners to return the dog to the shelter, and a survey of literature relating to dogs rehomed in a variety of developed countries has found that 7–19% were either returned to the shelter or given away [8]. 

Preventing aggression is therefore a major aim of shelter behaviour assessment, and past validations of the assessment methods have been concerned with testing the accuracy of prediction of aggression [9]. Standardisation of behavioural assessments for dogs in shelters is desirable to adequately assess their suitability for rehoming [10]. Currently there are several assessment schemes in place internationally. The Assess-A-Pet program and the SAFER (Safety Assessment for Evaluating Rehoming) test [11] both aim to provide the assessor with an understanding of a dog's likely response to a range of different stimuli post adoption, by challenging them in a number of situations and contexts within the shelter. A doll test correctly predicted 5 out of 7 cases of aggression towards children [11]. The main requirements are to assess aggression and fearfulness [12]. The 140-point SAFER test was validated using over 200 dogs in a series of telephone interviews with new owners. This test was good at predicting aggression and mouthing behaviour [11]. Another study [12] validated the predictive value of four tests used to assess the potential behaviours of aggression, fear, obedience, and separation anxiety by comparing the test results with the experiences of the new owners. They found that the tests predicted 74.7% of the potential problem behaviours correctly. 

We monitored a dog behaviour assessment used by the Royal Society for the Protection of Cruelty to Animals (RSPCA) in Queensland, and then repeated the assessment, with owner reporting of some items, in order to determine the repeatability of the different assessment components and the test's validity for predicting dog behaviour.

## 2. Materials and Method

Over a period of ten years (1995 to 2004) staff at the RSPCA shelter in Fairfield, Brisbane, Australia, developed a scheme for assessing dogs' behaviour to aid rehoming. All dogs over the age of four months were tested, and the assessment allocated a numerical score according to the behaviour displayed by the dog in response to a series of different stimuli. The total score on completion of the test determined whether the dog was eligible for rehoming. The assessment was terminated immediately if the dog displayed aggression to the handler (growling, baring teeth, or biting), in which case it was deemed to have failed the assessment. The assessment was also terminated immediately if the dog showed severe anxiety, evidenced by shaking, or avoided the assessor or the assessment tasks. In addition the dog failed if it escaped from the arena, for example, when it was left alone. 

Any two out of four RSPCA assessors (two males and two females) were randomly chosen to conduct each shelter assessment, observed by one researcher (AHP), who was trained until there was no difference betweenherscores and those of the shelter assessors. In the shelter assessment one RSPCA assessor administered the stimuli and the second recorded the dog's responses. In the second assessment, in the dog's new home, the trained researcher administered the assessment and the results were recorded by an assistant. Dogs arriving at the shelter were classified as sourced from local pounds with which the RSPCA was affiliated; surrendered by private persons; stray; seized by the RSPCA due to maltreatment or retrieved by the RSPCA ambulance. Dogs were housed at the shelter for any length of time from 24 hours to several months before they were deemed potentially fit for rehoming and therefore eligible for behavioural assessment. The assessment was planned to take approximately 20 minutes. 

The assessors initially monitored the dog's behaviour on entering an assessment arena from a standing position in the middle of the arena, noting any signs of caution in its behavioural response to the environment or the assessors, and any excitement/agitation, friendliness, or unsafe behaviours which might necessitate termination at that stage. The assessor did not engage in interaction with the dog at this time. Thereafter the dogs were subjected to 28 individual tests, described below, each of which had a numerical score from 0 to 5 (Table 1). These scores were designed to reflect the dog's state in response to the stimuli: a relaxed dog would receive a low score and an anxious or unsociable dog would receive a high score. 

The first two tests assessed how a dog responded to being touched by a stranger in a strange environment. The movements were rigid and there was a brief pause between each. The first test was three back strokes from head to tail, the second was five head pats and a score was allocated for the dog's response during the stimuli, during the pause in stimuli, and after the completion of the stimuli. The third test assessed the dog's tolerance of the assessor attempting to open the dog's mouth (five attempts of increasing vigour, each of 5 s). The fourth test was designed to simulate how the dog might be handled in a situation where it was examined by a veterinarian, and involved holding of the dog's ears, feet, tail, and body. The remaining tests were (5) holding the dog to simulate a hug for 15 s, with the response assessed during and after the hug; (6) rolling the dog over onto its back and holding it there for 15 s, with the response assessed during and after the roll; (7) presentation and retrieval, using an artificial hand, of a bowl of pet food and a piece of rawhide; (8) response to noise and movement (claps, hitting a metal bowl with a spoon, and the assessor jumping into the air, spreading arms and legs upon each repetition, three of each); (9) attempting to engage the dog in play using a rope toy, a tennis ball and a squeaky toy; (10) leaving the dog alone for 2 min; and (11) meeting the dog with two other dogs (one small and one large of different sex, if possible) both on and off the lead.

There were three possible outcomes for the dog:

Score 0–70 and no exhibition of severe anxiety, fearfulness or aggression: pass.
Score 71–80 and no exhibition of severe anxiety, fearfulness or aggression: placed on a behaviour modification program and then retested approximately one week later.
Score 81–135, or any exhibition of severe anxiety, fearfulness, or aggression: fail, in which case the dog was euthanased.

The behaviour modification program addressed undesirable behaviours exhibited by the dog at the time of its first assessment, and aimed to modify these behaviours before a second assessment took place. 

On the basis of their intimate knowledge of the dogs' behaviour, including that gained from the assessment, shelter staff made recommendations for each dog on the following: (1) whether they were suitable to enter a home with children, and if so, the appropriate age of children, whether the home needed to be surrounded by secure fences, (2) whether the new owner should be experienced at adoption, experienced with specific breeds, and (3) whether the dog was suitable for frail or elderly people.

### 2.1. External Influences on the Shelter Test

The behaviour assessments of 236 dogs (mean 30.2 months of age, SE 3.31 months) by the shelter staff were monitored from an office adjacent to the assessment arena between July 26 and September 30. The assessment arena measured approximately 6 × 6 m, was concrete floored and surrounded by a 1.6 m high steel fence. 

During each of the observations, the following were recorded for influence on the assessment result:

- duration of the assessment,
- number of people present in the area,
- defaecation by the test dog,
- presence of faecal deposits in the arena when the test dog entered,
- urination by the test dog,
- presence of urine in the arena when the test dog entered,
- presence of health and fitness defects, recorded by the assessor and a veterinarian prior to the assessment, and included a sore or missing body part; lethargy, docked tail, wound, dermatitis, matted hair; abnormal weight, recent parturition, in oestrus or particularly old,
- origin of the dog, classified as derived from the pound, privately surrendered, retrieved by the RSPCA inspectorate, picked up by an ambulance team,
- the presence of distractions during the test: people entering, leaving, or standing outside buildings adjacent to the assessment area, talking to the assessor or other people in the vicinity, walking past the assessment area, feeding animals in a field 3 m from the arena, using a vending machine outside an adjacent cabin, or the assessor answering a mobile phone.

### 2.2. The Repeatability of the Initial Test in the New Owner's Home

During the assessment observation period each of the 186 people adopting a dog from the shelter was asked by the reception staff to participate in a subsequent assessment, and 65 of these were recruited and signed a participant consent form. Five forms were invalid as they were signed by people who were adopting puppies that had not been behaviourally assessed, and 10 forms were incorrectly filled in. After a minimum of 65 days, 50 of these were contactable by telephone. Two people stated that they were no longer interested in participating in the study, two had passed the dog on to another person, one person had relinquished the dog to the RSPCA and one stated that their dog had been run over by a car. It was not possible to visit six of the 50 and phone interviews, which lasted a mean of 15 minutes, were used to administer a questionnaire on their satisfaction with their dog, detailed below. A total of 39 dogs that passed both the initial behavioural test and a medical assessment by the shelter's veterinarian were re-assessed in their new home after adoption from the shelter, and were the subject of the same questionnaire on satisfaction with their dog. 

The aim of the second behavioural assessment was to replicate the initial assessment in the dog's new home, using the researcher (AHP) as assessor, who had been trained to produce the same scores as RSPCA assessors. All of the behavioural assessments postadoption were carried out by two people, as in the first assessment, with one person administering the test (AHP) and the second recording the responses of the dog. The second assessment took place in either the back or front yard, in order to replicate as much as possible the outside environment of the initial assessment at the shelter. Both the assessor and assistant initially entered the house without making any contact or interacting with the dog in any manner. The assessments were then carried out in the centre of the area to replicate the assessment as it had been performed at the shelter. Other animals and people in the household, including the owner, were instructed not to be present during the assessment. Although it might have been more realistic to include the owner, it was felt that this would be a major confounding variable, as the dog's responses could be influenced by the owners' personality and behaviour. If distractions did occur from outside the perimeter of the area in which the dog was being assessed, the assessment continued at the same rate as the initial assessment at the shelter. The assessment lasted a mean of 10 minutes. 

Two of the initial tests were not conducted in the second assessment. The “Response when left Alone” test was not deemed appropriate in this environment as the owners had not left the home, and the “Response to other Dogs” test could not be included due to safety issues. In both cases, owners were asked about these issues in the postadoption questionnaire.

### 2.3. The Questionnaire

After the postadoption assessment had been completed, a questionnaire was administered verbally to the owner, and responses noted by the interviewer. Forty five owners were asked to rate, from one (least positive response) to five (most positive response) (1) how well the dog interacted with children; (2) how well they had bonded with the new dog; (3) how well the dog fitted in with the family and; (4) how satisfied they were with the dog overall. Owners were also encouraged to comment on their dog's behaviour. The owners' responses to the final two tests of the assessment (“Response when left Alone” and “Response to other Dogs”) were assessed by asking owners to estimate the most appropriate response from the options available in the first assessment. In addition, owners were asked if they had attended obedience training with the dog: how often they took the dog with them when they left the house and the dog's typical response to this situation: how much time the dog spent in the company of people, and how long the dog was walked each day.

### 2.4. Statistical Analysis

Statistical analysis was carried out using the SAS statistical system (version 8.2, 2001). Residual plots (normal probability plot, box and whisker plot, scatterplot and histogram) were used to test data sets for normal distribution. The association between the variable factors outlined earlier and the Part 1 test scores were analysed using chi-square tests of association (for categorical factors) or logistic regression (for continuous risk factors). For the continuous risk factors, such as the amount of time between first and second assessments, the assessment results were simplified to a binary outcome of pass (which included those animals which received behavioural modification) or fail, prior to analysis using binary logistic regression. In analysing the effect of the dogs' origin, an exact probability value was computed because of small numbers in some groups. In all other analyses, a likelihood ratio *χ* ^2^ test statistic was used to assess statistical significance. 

All of the dogs' responses to the first nine tests in the initial assessment at the shelter were measured against their responses to these tests postadoption using Kendall's tau-b coefficient, which is a measure of association that is nondirectional for use with binary or ordinal data. The Pearson correlation coefficient was used to compare final scores for the first and second assessments, and a paired *t*-test used to evaluate the change in score. 

The owners' ratings of their dogs' behaviour when meeting other dogs was not assigned a score and was not included in the data analysis, due to the differing and inconsistent nature between the initial and second assessments. In the initial test, dogs were assigned a score for this test based on their responses to a controlled sample of other dogs (usually one small and one large, one male and one female), both on- and off-lead. It was not possible to receive an accurate response from owners given these particulars and it was therefore deemed inappropriate to assign a comparable measurement for this test. The owner's questionnaire was also used to ascertain how the owners perceived their new dog and how the dog behaved when it was left alone, while in its typical environment in the home and when in the company of the owners. These results were analysed using basic statements about the tendencies of the dog and the owners' opinions about the dog.

## 3. Results

### 3.1. External Influences on the Assessment in the Shelter

Of the 236 dogs assessed, a total of 130 dogs passed, 82 dogs failed and 24 dogs were referred to a behaviour modification program. Reasons for failure are presented in Table 2. There was no effect of any specific health condition of the dog on the outcome of the assessment (pass, fail, or refer for a behaviour modification program). Health conditions were therefore amalgamated into one unit and this still had no effect on the outcome of the assessment (Table 3). The sex of the dog, whether it was neutered or entire at the time of assessment, and the dog's origin also did not have any effect on the outcome of the assessment. However, the presence of three or more faecal deposits in the arena at the start of the assessment increased the chance of the assessment dog passing the assessment and there was a tendency for the presence of urine to have the same effect. Defaecation or urination by the test dog did not affect the outcome. 

For the variables that were continuously distributed, only the duration of the assessment was positively correlated with the likelihood of passing the behavioural assessment (mean durations were 20.3 ± 1.00, 22.7 ± 0.51 and 26.1 ± 1.44 min for the Fail, Pass, and refer to Behaviour Modification Program outcomes, SED = 1.19, *P* = .005). There were no significant effects of time that the dog had spent at the refuge (mean durations were 7.2, 7.0, and 7.3 for the Fail, Pass and Behaviour Modification Program outcomes, SED = 0.82,* P* =.88), the number of people present (mean numbers were 4.8, 5.0, and 4.5 for the Fail, Pass and Behaviour Modification Program outcomes, SED = 0.25, *P* = .25) or the number of distractions (mean numbers were 3.7, 3.2, and 3.2 for the Fail, Pass and Behaviour Modification Program outcomes, SED = 0.52, *P* = .33).

### 3.2. Correlations between the First and Second Assessments

**Figure 1:**
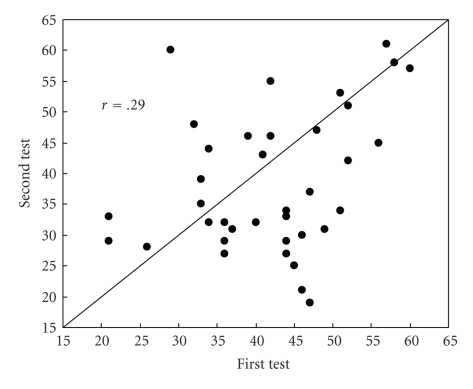
The relationship between the first and second test scores, with 1  :  1 line shown.

The mean time between each dog's first and second behavioural assessment was 80.9 d, SE 4.32, range 43–142 d. The correlation coefficient between the total scores on each of the tests was 0.29 (*P* = .08), with the score for the second assessment generally lower than the first (Figure 1), although this difference was not significant (*t* = 1.36, *P* = .18). There was no evidence of any relationship between the magnitude of change in assessment scores and the original total assessment score. Changes in score ranged from a decrease of 28 points to an increase of 31 points. 

There was no significant correlation between the first and second assessment scores in tests that involved direct contact with the assessor: back stroking, head patting, muzzle opening, touching ears, feet, tail and body and hugging (Table 4). There was a significant correlation between first and second assessment tests which did not involve direct contact between the dog and the assessor: food guarding, reaction to noise and movement, toys and play attempt. The dogs' responses after, but not during restraint were also correlated between assessments.

### 3.3. The Questionnaire

Only six of the 45 dogs whose owners completed the questionnaire had received obedience training. Two responses were removed from the survey. One of the people contacted by telephone had returned her dog due to nuisance barking and escape behaviour. This dog was recorded as being calm when left alone during the shelter behaviour assessment. Another dog had been passed on to a different family because it was too rough with the children in its adopting family. 

A total of 33 of the dogs had been or were regularly taken out of the house to take part in activities such as visits to the beach, a friend's house or on holidays, suggesting that the dog had become actively engaged in some of activities with their new owner. A total of 12 of these dogs were reported to display behaviours which indicated that they had been or continued to be anxious at such a time. The mean time spent in the company of people was 8 h/d and the mean time that each dog was taken for a walk was 27 min/d. 

A total of 40 owners responded that their dogs were generally calm in the presence of other people; 10 described their dogs as rough; 27 described their dogs as playful and one owner responded that their dog was aggressive (several owners responded that their dogs displayed more than one type of behaviour toward people other than themselves). All of the 45 dogs regularly interacted with children; 15 with old/frail people; and 2 with disabled people. Most dogs were calm or playful with people; ten had been rough with people, and one had been aggressive with a child. This was not deemed severe aggression by the adopter, as the dog was reported to “nip and snap” at a young girl in the family.

A total of 23 dogs were adopted into homes where another dog was already housed, and 14 dogs were adopted into homes that already housed at least one cat. A total of 27 dogs were described by their owner as friendly toward other dogs; 24 were playful; and 9 dogs had, on at least one occasion, been aggressive to at least one other pet in the household (some owners reported that their dog displayed more than one predominant behaviour towards other pets in the household). 

A total of 37 of the 45 owners responded that they were very satisfied with their new dog overall; 8 responded that they were satisfied. A total of 16 responded that they were very satisfied with how the dog responded to children in the household, 7 responded that they were satisfied with this, and 22 respondents did not have children in the household. A total of 35 owners responded that they felt very satisfied, and 10 were satisfied, with the way they had bonded with the dog. A total of 30 owners responded that they were very satisfied with the way the new dog had adjusted to the routine of the family and fit in overall and 15 responded that they were satisfied with this.

A total of 38 owners responded that the dogs liked to play with toys, while 7 responded that the dogs did not. Owners responded in detail to the final component of the questionnaire to express concerns about the behavioural idiosyncrasies exhibited by their adopted dogs. These included chewing (7 owners), barking (7 owners), and anxiety/fear (7 owners).

## 4. Discussion

### 4.1. External Influences on the Test

The presence of faeces and probably urine had a positive impact on a dog's likelihood to pass the behavioural assessment. As dogs communicate using the scent of their urine and faeces [12], it is likely that the excreta was perceived as the presence of another dog, producing a calming effect in this gregarious species and enabling higher scores to be achieved. The dog's own excreta had no such effect. The presence of a conspecific has a calming effect in many social species, for example, primates [13].

For most dogs admitted to shelters, cortisol levels rise significantly in the first 2-3 days [14] and then gradually decrease until day 9 [15]. Each dog that came into the RSPCA shelter was from a different background, which made it difficult for assessors to ensure that all dogs were at the same perceived stress level when they were behaviourally assessed. Consequently, dogs that were privately surrendered may have been at the shelter for as little as 24 hours when they were behaviourally assessed, or as long as four or five days. Dogs that had been seized from their owners due to maltreatment may have been at the shelter for many months before being behaviourally assessed. In Queensland, stray dogs are required by law to be at the shelter for at least seven days before they can be classed as unclaimed, become the property of the RSPCA, and proceed through the processes leading to adoption. These significant variations in the times that dogs had been at the shelter, however, did not have an impact on the outcome of the assessment. 

The majority of distractions occurring during the behavioural assessments, caused by people or otherwise, had no impact on a dog's likelihood to pass or fail the behaviour assessment. There were many events that took place around the assessment area and dogs often appeared distracted by them, as they turned their heads to see or walked over to investigate the nature of the distraction while the behavioural assessment was occurring. This appeared to result in the dog responding in an uncharacteristic manner to the stimuli offered during the assessment. It is possible that this occurred, as a dog received a higher score if it was disinterested, for example, in the toys presented during this test of the assessment, but the assessors may have been aware of the potential impact of these distractions on the dog's behaviour and adjusted the score accordingly.It was not possible to assess, in this study, how a dog might otherwise have reacted to stimuli had it not been distracted in certain tests of the assessment.

The correlation between the duration of the assessment and the likelihood that a dog would fail was anticipated, because the length of the behavioural assessment was directly dependent on the responses displayed by the dogs. If a dog was aggressive, excessively anxious, or the behavioural assessor felt too uncomfortable to continue, the behavioural test was terminated. Conversely, if a dog continued to respond to the stimuli with behaviours that were either desired or not immediately of concern to the assessor, then the assessment continued. However, the greatest difference in duration observed was between Pass and Refer for Behavioural Modification categories (neither of which involved premature termination of the test), which probably relates to some prolongation of tests in the event as a result of uncertainties in the latter category.

### 4.2. Assessment Results

Shelters elsewhere, particularly in the United States, focus on using their behavioural assessments to identify aggressive dogs and dogs with aggressive tendencies to detect those which are likely to become aggressive postadoption [7]. The Fairfield shelter adopted a similar no-tolerance approach for aggression, but it also aimed to euthanase dogs if they were deemed too anxious or fearful to be considered suitable for rehoming. A total of 33 dogs failed the behavioural assessment as a result of fearfulness or anxiety-related behaviour, the second highest reason for failing the assessment. A further 12 dogs that were successfully rehomed and whose owners had completed the questionnaire postadoption had been anxious when they were taken outside the home to partake in activities. Some anxiety is to be expected in situations such as these, even when the dog is in the company of the family. While dogs can fail the behavioural assessment immediately for exhibiting such behaviours, the assessment is structured in such a way that dogs which are anxious or fearful will accumulate a score that deems them unsuitable for rehoming. As evidence of the desirability of this, seven of the new owners cited anxiety or fear as a problem in their dogs. However, anxiety is not a major reason for relinquishment [1], suggesting that it may be a transient problem confined to the dog's time in the shelter and the immediate period after rehoming. Significant stressors in shelters include a high level of noise, novelty, social isolation, and prolonged confinement [16]. Minimising the stressfulness of the assessment arena by making it more like a living room has been advocated [14]. 

Of the 236 dogs observed, 20 dogs failed the behavioural assessment due to aggression towards other dogs. However, of the 45 owners who adopted dogs that had passed the test and who took part in the questionnaire postadoption, 10 reported that their dog had been aggressive towards at least one other dog, demonstrating an inability of the test to adequately predict aggressive tendencies towards conspecifics. Aggression is the single most common cause of dog return to shelters [4]. A dog's tendency to be aggressive toward other dogs is thought to be an innate behaviour [17] and a much longer (1.5 h) behaviour assessment has been shown to correctly identify potentially aggressive dogs [12]. However, it is a difficult trait to determine from a dog's behaviour [18, 19], with different tests having low levels of reliability and hence validity [20]. The best indicators are a low posture in the dog [21] and the absence of playfulness [22]. Training dogs in the shelter to be good with other dogs increases the chance of their being rehomed [23], but their behavioural responses to other dogs are difficult to assess consistently [24]. It is therefore possible that the assessment that was used by the RSPCA in Fairfield did not sufficiently challenge the dogs to display aggression towards other dogs. 

Eleven dogs failed the behavioural assessment as a result of exhibiting escape behaviours or vocalizing excessively when left alone. The problem of barking dogs and dogs which stray in the community is a concern for any council as well as for the owners [25]. Therefore it is deemed inappropriate for the RSPCA to rehome dogs which exhibit such problem behaviours. Of the dogs that passed the behavioural assessment and were rehomed, seven were reported by their owners to have escaped the confines of their backyard either by digging under the fence or jumping over it. Seven dogs had also vocalized excessively postadoption and two of the owners had deemed this vocalization excessive enough to purchase anti-barking electronic shock collars for their dogs. These are behaviours which would warrant an immediate fail if displayed by the dog during the initial behavioural assessment at the shelter. There was no correlation between the first and second assessments in response to being left alone, which may partly reflect the fact that it was tested empirically in the first test but by questionnaire in the second. Improved methods of detecting escape tendencies in the dogs need to be developed.

### 4.3. Comparing the Shelter and Postadoption Assessments

The lack of correlation between the shelter and postadoption assessments for tests in which direct contact with the assessor occurred suggests that the dogs had either specific responses to individuals or characteristics of the individuals at the time. They may have been assessing subtle behaviour signals that were undetectable to the observer (AHP) that was common to both sets of assessments. Correlations between and within scientists and handlers in their subjective assessment of dog's ethological characteristics are usually high [26, 27], but there is only moderate consistency between shelter staff in their ability to assess these characteristics [24]. Although formal tests of intra and interobserver reliability were not conducted in this study [2, 28], but would be desirable in a more detailed study, the researcher (AHP) trained with shelter staff until their scores were identical. The long-time interval between tests may have reduced the correlation between the two: a retest of a similar suite of behavioural measures that was conducted after just 40 days gave a higher correlation (*r* = 0.58), than our test (*r* = 0.29) conducted a mean of 81 days later [3]. 

It is possible that responses to novel people in the home environment were fundamentally different to those in the shelter, where the dogs could be confronted with unfamiliar people regularly. Responses tended to be decreased in the second assessment, although not significantly, which could indicate greater relaxation in the home environment. In the shelter environment dogs may perceive themselves more as pack animals with greater emphasis on preserving their position in the dominance order. In the home environment their position is fixed by the usually dominant position of other humans in the domain and it is stable. Thus their tolerance of human interaction may be context specific. 

The responses to hugs were not correlated between the two tests, whereas those after restraint were. The benign nature of the hug probably did not leave any lasting impact, and in contrast the restraint was probably a more adverse experience. Restraint is a test widely used by behaviourists to assess the temperament of puppies and is likely to be an innate response, depending on the dog's acceptance of humans. In chickens the response to restraint during bodily inversion is genetically controlled, with the microsatellite for this trait having been identified [27]. 

Dogs' responses to being given food during the “Food Guarding” test were correlated between first and second assessments. Dogs are opportunistic carnivores and an innate response to readily take food when offered is likely to be adaptive. The nutritional environment was likely to be similar in the two situations, since dogs are usually fed in accordance with food manufacturer's recommendations in both shelters and in the home. Noise and movement responses were also correlated. Responses to these stimuli are likely to be adaptive, and noise at least is known to be a significant stressor in dog kennels [16]. Similarly, the response to play and toys was strongly correlated between the two tests.

### 4.4. The Questionnaire

Only six of the 45 owners who partook in the questionnaire had taken their dogs to obedience training, even though it is recommended by RSPCA Fairfield that dogs should complete basic obedience training with their owner after adoption. This could be responsible for the high level of owners reporting concerns about behaviour problems displayed by their dogs. Nevertheless, the level of return of dogs to the shelter in this study (5%), albeit over a period of just 81 d, was less than the 7–19% that are usually returned to shelters [6]. Escape behaviour was commonly reported in the questionnaire, which is probably due to the living style in Brisbane where many dogs are kept outdoors all the time. Fences are often insecure or too low to contain the dog and there are many enticements to escape, such as the opportunities for social contact with other dogs.

The reporting of such behavioural problems in the questionnaire may indicate that dogs with a tendency for behavioural problems did not show these at the shelter, and the shelter behavioural assessment was therefore inadequate in its identification of current and potential problems. It could also indicate that dogs are accepted into families and valued in spite of their problems or it could indicate that many dogs develop problem behaviours soon after being rehomed because their owners are either neglectful or reinforce negative behaviours. Finally, it could indicate that the dog's typical behaviours were not displayed in the atypical environment of the shelter. The owners' acceptance of these behaviours, which were not identified at the shelter during the behavioural assessment, suggests that the shelter could relax the criteria for passing the behavioural assessment and successfully continue to rehome dogs.

## Figures and Tables

**Table 1:** Criteria for the scoring system.

Score	Criteria
0	Active and positive engagement with the assessor or a positive response to the stimuli introduced
1	Relaxed or passive response
2	Acceptance after a few attempts and eventual tolerance
3	Overexcitement
4	Dog was attempting to avoid the assessor or the stimuli or used mouthing
5	Exhibition of fear, tonic immobility, or escape from the arena

**Table 2:** Reasons given by assessors for failing dogs during the initial behaviour test (more than one reason was given for some dogs).

Reason	Number of dogs
Fearfulness	22
Aggression toward humans	11
Aggression toward other dogs	20
Untrustworthiness	9
Dog too forward or rough	8
Resource guarding	5
Excessive vocalization/escape behaviour	11
Total score too low	10

**Table 3:** The effects of health problems, gender, neutered status, origin, faeces and urine from the previous dog test and defaecation and urination during the test on the number of dogs in the pass, fail and behaviour modification program (BMP) categories of the first test.

	Test outcome (n)				
Variable	Fail	Pass	BMP	%Failure	Probability
Health problem					
Yes	29	43	10	35%	0.72
No	53	87	14	34%	
Gender					
Male	42	63	16	35%	0.25
Female	40	67	8	35%	
Neutered					
Yes	16	35	6	28%	0.46
No	66	95	18	37%	
Origin					
Pound	11	22	5	29%	0.92
Surrendered	37	57	9	36%	
Stray	25	39	8	35%	
Ambulance	5	9	2	31%	
Inspectorate	4	3	0	57%	
Faeces from previous dog					
Absent	28	39	8	37%	0.04
One	30	35	6	42%	
Two	17	26	2	38%	
Three or more	7	30	8	16%	
Urine from previous dog					
Present	50	93	7	33%	0.19
Absent	32	37	16	38%	
Defaecation during test					
Yes	54	91	17	33%	0.50
No	28	39	8	37%	
Urination during test					
Yes	50	93	17	31%	0.56
No	32	37	7	42%	

**Table 4:** Mean scores, Kendall tau B values and probability of significant correlation for the different tests of the first (shelter) and second (home) assessments.

	Test scores		Kendall's tau-b	Probability
	Shelter	Home	value	
Back stroking				
During strokes	1.51	1.67	0.12	.39
In between strokes	1.74	1.41	0.03	.80
After strokes	1.92	1.92	0.14	.33
Total	5.18	5.00	0.08	.50
Head patting				
During pats	1.69	1.33	0.09	.54
In between pats	1.64	1.64	−0.02	.89
After pats	1.87	1.87	0.02	.88
Total	5.21	4.82	0.07	.58
Muzzle touch tolerance	2.56	2.67	0.23	.10
Touching ears, feet, tail, and body	8.18	9.15	0.10	.42
Hug				
During hug	1.90	1.82	0.24	.09
After hug	2.44	2.18	−0.21	.12
Total	4.33	4.00	−0.08	.53
Restraint				
During restraint	2.44	2.38	0.14	.32
After restraint	2.46	2.18	0.37	<.01
Total	4.87	4.56	0.25	.05
Food guarding	3.03	1.95	0.32	.02
Reaction to noise and movement	4.87	3.41	0.26	.03
Reaction to toys and play attempt	3.67	3.56	0.49	<.01
Reaction to being left alone	1.07	0.73	−0.14	.37
